# Comparing Foodie Calls in Poland, the United Kingdom, and the United States: A Registered Replication Report

**DOI:** 10.1177/00332941231164079

**Published:** 2023-03-16

**Authors:** Mehmet A. Orhan, Brian Collisson, Jennifer L. Howell, Marta Kowal, Thomas V. Pollet

**Affiliations:** 88546 EM Normandie Business School, Clichy, France; 5995 Northumbria University, Newcastle Upon Tyne, UK; 166699 Azusa Pacific University, Azusa, CA, USA; 33244 University of California Merced, Merced, CA, USA; 49572 University of Wroclaw, Wroclaw, Poland; 5995 Northumbria University, Newcastle Upon Tyne, UK

**Keywords:** Dating, food, dark triad, gender roles, replication, preregistration

## Abstract

Collisson et al. (2020) found Dark Triad traits and gender role beliefs predicted “foodie calls,” a phenomenon where people go on a date with others, to whom they are not attracted, for a free meal. Because gender roles and dating norms differ across cultures, we conducted a registered replication across different cultures by surveying 1838 heterosexual women from Poland, the United Kingdom (UK), and the United States (US). Relying on the structural equation modeling, as conducted in the original study, our findings revealed gender role beliefs best predicted foodie calls and their perceived acceptability, whereas the Dark Triad’s general factor was nonsignificant. Analyses at the country level yielded mixed results. The original findings were replicated in the UK and Poland, but not in the US, where only narcissism predicted foodie calls. In the US, gender role beliefs predicted foodie call acceptability, but the Dark Triad general factor did not. Potential reasons for why traditional gender roles, but not the Dark Triad, predicted foodie calls in the US are discussed.

## Introduction

Dating can be expensive. Given rising inflation, supply chain issues, and other costs associated with in-person dining (Swanson, 2022), the cost of going out to eat adds up. According to the Food & Agricultural Organization’s 2022 global food price index, food prices are nearing record highs. Indeed, the United States Department of Labor reports the average cost of dining out climbed 6% since 2021, the largest single-year increase since 1982 (Lucas, 2022). Given the financial investment in dating, people may ask who should pay for a meal on a first date.

Cross-cultural differences in dating norms often inform which partner is responsible for paying for food on a date. In societies where traditional gender roles are common, such as the United States, men are usually expected to pay the bill (Delucia, 1987; Jaramillo-Sierra & Allen, 2013; Wu et al., 2021). In fact, a Spanish phrase common in South America, “pagar a la americana” or “to pay American style” refers to a Spanish man paying the total cost of a meal for himself and his date (Going Dutch, 2022). Whereas in more egalitarian societies, with less explicit traditional gender role beliefs (Eaton & Rose, 2011), dating partners may be expected to share or split the cost of a meal. Colloquial phrases, such as “going Dutch” and “pagare alla Romana” (to pay as they do in Rome), highlight cultural differences in who is expected to pay for a date, such as sharing the cost of food equally. Still, traditional dating scripts amongst heterosexual couples suggest when a man asks a woman to go on a date, it is assumed the man incur the cost of the meal and the woman’s acceptance of the invitation suggests at least a modicum of potential romantic interest (Delucia, 1987; Jaramillo-Sierra & Allen, 2013; Wu et al., 2021).

In 2020, Collisson et al. conducted two online surveys of heterosexual women in the United States regarding whether they had ever engaged in a *foodie call*, “a situation when a person, despite a lack of romanticattraction to a suitor, chooses to go on a date to get a free meal” (*p*. 425). They measured how frequently heterosexual women engaged in foodie calls. They found between 23% and 33% of American women had engaged in a foodie call. Of those that had done so, approximately 25% did so frequently or very frequently. The majority of women overall rated foodie calls as extremely or moderately unacceptable.

Notably, in Study 1 of their article, Collisson et al. found individual differences in traditional gender role beliefs and Dark Triad traits predicted women’s likelihood of engaging in foodie calls. Women who scored highly on traditional gender role beliefs, as well as those who scored high in the Dark Triad - a constellation of three antisocial personality traits: narcissism, Machiavellianism, and psychopathy (Paulhus & Williams, 2002) - were more likely to engage in foodie calls and perceive them as acceptable. Specifically, narcissistic women, who tend to be self-focused, entitled, and lack interest in warm relationships (Campbell & Foster, 2007), Machiavelli women, who tend to be especially cunning, manipulative, and deceptive (Miller et al., 2017), and women high in subclinical psychopathy, who presumably lack empathy and remorse, were assumed most likely to engage in foodie calls. Collisson et al. found narcissism, Machiavellianism, and psychopathy each correlated with whether women had engaged in a foodie call and its perceived acceptability. Yet only the general Dark Triad factor, not the orthogonal traits, predicted whether women had engaged in a foodie call and its perceived acceptability. In addition to assessing the relation of the Dark Triad, Collisson and colleagues also reported traditional gender role beliefs predicted whether women had ever engaged in a foodie call. In estimating the acceptability of foodie call behavior, the general constellation of the Dark Triad traits appeared to be the only significant predictor of foodie call acceptability.

As the original study did not entail a representative nor cross-cultural sample, the impact of gender role beliefs and personality traits can be overlooked. In this study, we aim to test these effects in different cultural contexts and replicate the original findings of Collisson et al. (2020, Study 1) in order to test their generalizability.

### Traditional Gender Role Beliefs and Cultural Dating Norms

According to social role theory (Eagly, 1987), men and women tend to espouse normative roles regarding how men and women should act. Such gender role beliefs reflect societal expectations and refer to “gender-related tasks and power distribution” (Arends-Tóth & van de Vijver, 2007, *p*.813). Although men and women often behave similarly in many social settings, stereotypical gender roles are commonly seen in dating situations (Lever et al., 2015; Vogel et al., 2003). Within dating contexts, people who espouse traditional gender roles expect men to initiate courtship, act chivalrous (e.g., open a door or hold a chair for a woman), and cover expenses associated with the date (Delucia, 1987; Jaramillo-Sierra & Allen, 2013; Wu et al., 2021).

However, gender roles may vary across individuals, cultures, and time. For instance, women’s increasing participation in the workforce, particularly in economically advanced societies, may challenge traditional gender roles and assumptions (Boehnke, 2011; Lever et al., 2015). Additionally, gender equality movements throughout the world may make men and women more likely to share costs associated with dating (Gillespie et al., 2019), especially in Western societies (Eaton & Rose, 2011; Rudman & Glick, 2021). Based on a study with nearly 20,000 American, heterosexual, unmarried participants, Lever et al. (2015) found a majority of men and women share the expenses in dating situations, and around two-thirds of men and approximately half of women consider equality in cost-sharing as the desired outcome.

Gender role beliefs are not independent of cultural norms. Arends-Tóth & van de Vijver (2007) noted culture plays a key role in perception of gender roles separation and the acceptance of these roles within a particular cultural context. Distinct roles of men and women are often context-dependent and influenced by culture, family, personal experiences, and social interactions (Eagly, 1987). In inherently hierarchical societies, roles of men and women tend to comply with traditional gender views (Arends-Tóth & van de Vijver, 2007). Men are assumed to act assertively, dominantly, and bear responsibility, both physically and financially, for the safety and security of their partners; whereas women are assumed to act passively, subservingly, and bear responsibility as caregivers (Arends-Tóth & van de Vijver, 2007). When gender roles are clearly defined in society, and members widely accept the norms associated with these roles, men are expected to pay the cost of the meal when dating. Traditional gender roles and associated dating scripts may be rife opportunities for women who score high on the Dark Triad to engage in foodie calls. In the current study, we suspect foodie calls and their acceptability may be culturally dependent and vary to the extent to which women espouse traditional gender role beliefs and Dark Triad traits.

### Current Study

Although Collisson et al.’s (2020) foodie call study revealed the prevalence of foodie calls in two samples of heterosexual women, and linked foodie calls to traditional gender role beliefs and Dark Triad traits, it was limited in several important ways. First, its findings were drawn from women residing in the United States. Second, its findings were nuanced, such that a Dark Triad general factor, rather than the orthogonal traits, emerged as the predictor of foodie call behavior and acceptability. Given the importance of replication in psychological science (see Klein et al., 2014), particularly collaborations involving original authors (Klein et al., 2022), and the need to explore dating phenomenon cross-culturally, we conducted a registered replication and extension of (Collisson et al., 2020, Study 1) by surveying heterosexual women from Poland, the United Kingdom (UK), and the United States (US) - cultures associated with varying gender role beliefs and dating scripts - regarding foodie calls and their perceived acceptability. Considering the monocultural setting of the original study, this study was designed to understand the impact of cultural contexts. To ensure the generalizability of the results, there is a need to cross-validate the hypotheses for different social settings. As culture plays a significant role in accepted norms and behaviors of men and women, it may be uncertain if the personality traits standalone could explain the behavior and its acceptability. Ultimately, the acceptability of such behavior can be context dependent.

The current study compares women from Poland, the UK, and the US. Prior research reveals nation-level differences in perceived gender equality and roles (Stavrova et al., 2012). In fact, variations in economic conditions and female workforce participation rates may influence societal views on gender roles, such that when countries develop economically, gender role beliefs tend to be more egalitarian (Boehnke, 2011). Poland, UK, and the US represent three countries with divergent economic conditions, female workforce participation, and potentially different gender roles. According to the Gender Inequality Index published by the United Nations Development Programme (2020), the US has historically had more gender inequality than Poland and the UK, exhibiting almost identical trends since 1995. In regard to economic indicators, the US and UK show similar trends in female workforce participation rates and Gross Domestic Product (GDP) per capita figures, whereas Poland is developing economically (The World Bank, 2022). Such cultural differences, and the authors’ access to participants, make comparing foodie calls and their predictive traits among women from Poland, the UK, and the US worthwhile.

As culture plays a significant role in accepted norms and behaviors of men and women, it is unclear if Dark Triad traits alone explain foodie calls and their perceived acceptability. Perhaps gender role beliefs, which are culturally-dependent, may better predict foodie calls. Therefore, to replicate and extend Collisson et al. (2020), we conducted a pre-registered replication. The protocol, including questionnaires, sample size, and data analysis plan, was pre-registered on Dec 12, 2021, and is publicly available on the Open Science Framework (OSF) website: https://osf.io/mw3n4/?view_only=9c63a976f2a841458a6715b847435a39. The questionnaires were prepared in three different languages; Polish, Turkish, and English (see Method for more details).

## Method

### Sample Calculation, Power Analysis, and Participants

During pre-registration, we determined the required, highly powered sample size based on the following formula for successful replication: Sample size = 2.5*Original study sample size (678) = 1,695, as suggested by Brandt et al. (2014) and Simonsohn (2015). A clustered sampling method was applied, and the minimum sample size per country was set at 250 respondents. Initially, four countries, Poland, the UK, Turkey, and the US, were selected for data collection, but we dropped the Turkish site because we did not receive sufficient responses to meet the 250 respondent criteria. Consequently, three countries were retained to conduct the analysis; Poland, the UK, and the US. In alignment with the original study (Collisson et al., 2020; Study 1), only heterosexual women were recruited from the three countries using two online panels, including Amazon MTurk and Prolific, as well as from the researchers’ own extended network. The remuneration payouts for completing the survey for each participant ranged from $0.26 to $0.58. These variations in the remuneration were due to the constraints each panel imposes. The minimum pay restriction imposed by Prolific was approximately $8/hour, whereas MTurk did not enforce any limit.

Participants who did not comply with the required sample characteristics related to gender and sexual orientation and those who failed the attention check were excluded from the study. The number of excluded cases was 273. We retained the data from 1838 valid submissions of heterosexual women completing our survey. Responses of 547 women from Poland, 308 from the UK, and 983 from the US were recorded. The mean age of the entire sample is 33.70 (*SD* = 12.70). The Polish sample has an average age of 25.6 (*SD* = 7.20). The average ages are 35.9 in the British sample (*SD* = 12.18) and 36.32 in the American sample (*SD* = 14.35). Polish participants’ mean age is significantly lower than the UK sample (*t* = 13.40, *df* = 423, *p* = .000) and the US sample (*t* = 23.01, *df* = 1443, *p* = .000), whereas the difference between the US and the UK is not significant (*t* = .40, *df* = 452, *p* = .914).

### Materials

#### Foodie Call Frequency

As in the original study, we asked participants a yes/no question to assess whether they had previously engaged in a foodie call behavior. The question read, “Have you ever agreed to date someone (who you were not interested in a relationship with) because he might pay for your meal?” We also asked the frequency on a 7-point Likert scale ranging from 1 (*never*) to 7 (*always*). Additionally, we also asked the number of past behavior if a woman had engaged in such behavior in an open-ended question.

#### Foodie Call Acceptability

In line with the original measure, the acceptability was assessed on a 7-point Likert-type scale (1 = *extremely unacceptable* to 7 = *extremely acceptable*; midpoint 4-neutral) with the following question; “How acceptable do you think it is to date someone (you were not interested in a relationship with) because he might pay for your meal?”

#### The Dirty Dozen Dark Triad

We used the shortened version of the Dirty Dozen Dark Triad (DDDT), which consists of 12 items for three dimensions of dark personality (Jonason & Webster, 2010). Each dimension, Machiavellianism, psychopathy, and narcissism, contained four items. For the Polish version, we used the validated translation by Czarna et al. (2016). The DDDT was also measured on a 7-point Likert-type scale ranging from 1 (*strongly disagree*) to 7 (*strongly agree*).

#### Traditional Gender Role Beliefs

The same measurement items were used again. The 10-item, shortened version of the Gender Role Beliefs Scale of Kerr and Holden (1996) was used as suggested by Brown & Gladstone (2012). This construct was also measured on a 7-point Likert-type scale ranging from 1 (*strongly disagree*) to 7 (*strongly agree*).

#### Control Variables

##### Demographic variables

We directed several questions in regard to the demographic characteristics of the sample. These questions included age, education, employment, and relationship status. For the education variable, we slightly modified the education levels in each country to account for the differences in educational systems. Then we re-coded them on a 6-point scale; 1 (less than high school), 2 (high school graduate), 3 (some college but no degree), 4 (associate degree), 5 (undergraduate degree), 6 (professional and postgraduate degrees). We also recoded the education variable in the original dataset to make the comparisons more meaningful. The comparison of descriptive statistics per country is provided in Table 3.

##### Other variables

Additionally, we asked if they had ever used an online dating platform and if yes, we asked them to specify the names of platforms used. Finally, we enquired about their perceived self-attractiveness to test the mediating impact on the relationship between dark personality and foodie call behavior and its acceptability.

#### Translation of Surveys

In Table 1, we present descriptive statistics, reliabilities, and correlations between each construct measured in our study. In addition to the reliability scores of each construct for the pooled dataset, we provide the Cronbach’s alpha scores at the country level in Table 2.

**Table 1.** Descriptive Statistics and Correlation Matrix.

Measure	α	*M*	(*SD*)	2	3	4	5	6
1. Previous foodie call	—	.34	(.47)	.53*	.49*	.54*	.44*	.50*
2. Foodie call acceptability	—	3.22	(1.91)	—	.58*	.61*	.54*	.52*
3. Machiavellianism	.91	3.57	(1.74)		—	.87*	.72*	.65*
4. Psychopathy	.92	3.17	(1.81)			—	.69*	.76*
5. Narcissism	.90	4.17	(1.64)				—	.55*
6. Gender role beliefs	.92	3.35	(1.46)					—

*Note*. *N* = 1838. Previous foodie call is a dichotomous variable (no = 0, yes = 1), so the correlation is a pearson point biserial. *p < .001

**Table 2.** Reliability Scores of All Study Constructs per Country.

Measure	α_PL_	α_UK_	α_US_	α_ORIGINAL STUDY_
Machiavellianism	.84	.85	.92	.87
Psychopathy	.75	.76	.94	.88
Narcissism	.86	.87	.91	.89
Gender role beliefs	.80	.85	.91	.87

For the English version, we relied on the measures as assessed in the original study. On the other hand, the Polish version was a translated version. It was first translated automatically and then corrected and cross-validated independently by two professional translators. The final version was checked by another native speaker who works as a psychology researcher at a Polish university.

### Procedure

The questionnaires in Polish and English languages were posted online on Amazon MTurk and Prolific. We made the selection criteria explicit in the advertisements that only heterosexual women from Poland, the UK, and the US would be recruited. The questionnaire started with qualifying questions that asked participants their gender and sexual orientation. Those who did not identify as ‘female’ and ‘heterosexual’ were not allowed to proceed further. Then, all participants completed the foodie call, Dark Triad, and traditional gender role belief measures in randomized order. All materials, both with Polish and English versions, are available on the OSF Web site as well: https://osf.io/mw3n4/?view_only=9c63a976f2a841458a6715b847435a39.

## Results

### Analyses

For the analyses, we utilized multiple resources. The initial analyses on descriptive statistics and reliability scores were conducted using IBM SPSS Statistics 27. To run the structural equation models, we relied on the same syntax as in the original study. Based on the codes, we derived the full model using the conceptual depiction of the study, as shown in Figure 3. Using the SEMLj syntax v. 0.8.0 module, which is run based on the lavaan package, we tested our models on jamovi’s latest version, 2.3.12.0. The analyses for the pooled data and for each country can also be found under the same OSF link: https://osf.io/mw3n4/?view_only=9c63a976f2a841458a6715b847435a39.

**Table 3.** Comparison of Descriptive Statistics.

Measure	Country	N (Missing)	M (SD)	Median	Min	Max
Previous foodie call (*No* = 0, *Yes* = 1)	PL	547	.14 (.34)	0	0	1
	UK	308	.07 (.26)	0	0	1
	USA	983	.53 (.50)	1	0	1
	ORIGINAL	678	.23 (.42)	0	0	1
Foodie call acceptability	PL	547	2.85 (1.57)	3	1	7
	UK	308	1.95 (1.39)	1	1	7
	USA	983	3.83 (1.98)	4	1	7
	ORIGINAL	674 (4)	2.68 (1.79)	2	1	7
Machiavellianism	PL	547	2.89 (1.35)	2.75	1	7
	UK	308	2.60 (1.27)	2.50	1	7
	USA	983	4.25 (1.77)	4.75	1	7
	ORIGINAL	673 (5)	2.99 (1.58)	2.75	1	7
Psychopathy	PL	547	2.20 (1.08)	2.00	1	6.25
	UK	308	1.82 (1.01)	1.50	1	7
	USA	983	3.90 (1.99)	4.50	1	7
	ORIGINAL	673 (5)	2.31 (1.54)	1.75	1	7
Narcissism	PL	547	3.92 (1.43)	4.00	1	7
	UK	308	3.12 (1.36)	3.00	1	7
	USA	983	4.63 (1.64)	5.00	1	7
	ORIGINAL	673 (5)	3.51 (1.57)	3.75	1	7
Gender role beliefs	PL	547	2.38 (.80)	2.20	1	5.30
	UK	308	2.38 (.91)	2.20	1	6.50
	USA	983	4.19 (1.37)	4.40	1	6.50
	ORIGINAL	674 (4)	3.27 (1.25)	3.30	1	6.50
Age	PL	542 (6)	25.64 (7.20)	23	17	63
	UK	303 (5)	35.87 (12.18)	34	18	77
	USA	967 (22)	36.18 (10.49)	34	19	78
	ORIGINAL	669 (9)	35.46 (11.09)	32	18	69
Education	PL	545 (2)	3.31 (1.28)	3	1	6
	UK	306 (2)	3.71 (1.05)	4	1	6
	USA	978 (5)	4.01 (.78)	4	1	6
	ORIGINAL	669 (9)	4.30 (1.30)	5	1	6

### Descriptive Statistics

Table 3 shows the means, standard deviations, medians, minimum and maximum scores of each study construct, and selected demographic variables (i.e., age and employment status) per country in contrast to the original study’s dataset which is available on https://osf.io/zxqwd. According to the results of our study, previous foodie calls were reported much more frequently in the US sample. Out of 983 US women, 52.8% admitted that they had previously engaged in a foodie call. The lowest frequency of past behavior was reported by respondents in the UK, where only 7.14% admitted so (*n* = 22). Likewise, its acceptability was the highest in the US (*M* = 3.83, *SD* = 1.98). These acceptability ratings are found to be significantly higher than the original observations in the US (*t* = 12.1, *p* = .000, *d* = .60). Moreover, in our dataset, we also found that the current US sample’s all three dimensions of the Dark Triad personality traits were reported higher than they were reported in the original study. The UK sample, however, had the lowest scores in all these three personality dimensions. Finally, according to the participants’ self-reported gender role beliefs, the US sample (*M* = 4.19, *SD* = 1.37) was found to be more traditional than the Polish (*M* = 2.38, *SD* = .80) and UK samples (*M* = 2.38, *SD* = .91).

Moreover, we assessed whether country-level mean differences of study variables are statistically significant. Our results indeed confirm that all measures, previous foodie call behavior, its acceptability, the Dark triad personality characteristics statistically differ based on country. The details of these analyses in ANOVA analysis file under the same OSF link, where all other analyses are publicly available.

### Frequency of Foodie Calls

**Figure 1.**
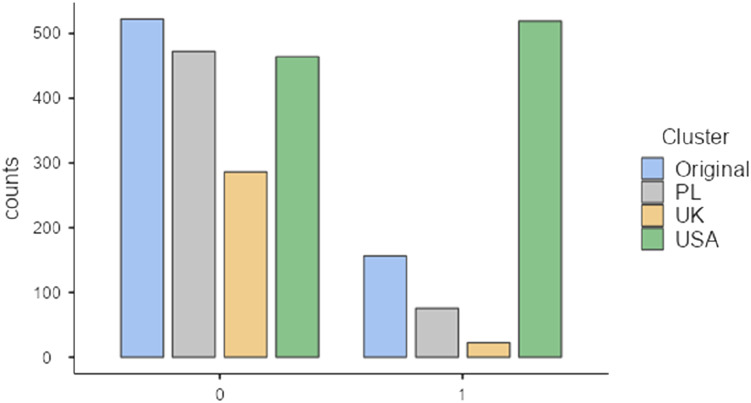
Previous foodie call behavior (No = 0, Yes = 1).

In our sample, most participants (66.49%) reported that they did not engage in a foodie call behavior in the past (*n* = 1222). However, 616 of them reported that they had indeed previously engaged in a foodie call. Of those who admitted to doing so, the large majority of them (84.25%) come from the US sample (*n* = 519). Surprisingly, the US was the only sample in which the number of respondents who engaged in the foodie call behavior exceeded the number of those who did not commit this (*yes* = 52.8%, *no* = 47.2%). Of 547 Polish women, 13.07% (*n* = 75) reported engaging in a foodie call. On the other hand, the lowest frequency of foodie calls was observed in the UK. Only 7.14% (*n* = 22) of 308 respondents from the UK reported that they did engage in such behavior. The country-level comparison of the past foodie call behavior is shown in Figure 1 and Table 4.

**Table 4.** Frequencies of Foodie Call Behavior.

Foodie Call Behavior	Cluster	No	(%)	Yes	(%)	Total (*N*)
Original study	USA	522	76.99	156	23.01	678
Current study	PL	472	86.93	75	13.07	547
	UK	286	92.56	22	7.14	308
	USA	464	47.20	519	52.80	983
Total of current		1222	66.49	616	33.51	1838

**Figure 2.**
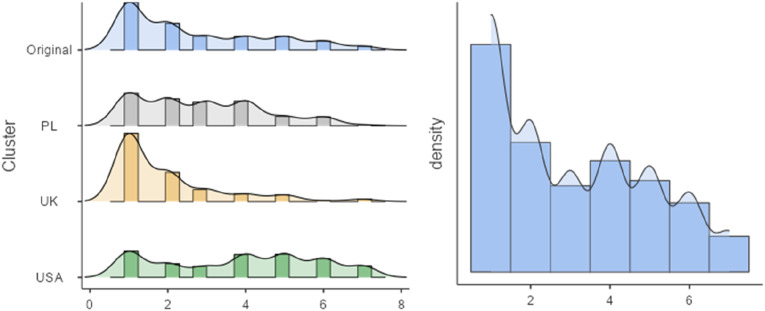
Frequency distribution of foodie call acceptability. Note: The right graph presents the histogram of the pooled data collected in the current study.

As was the case in the original study, the distribution of the acceptability data is positively skewed, such that the majority of women (55.39%) found this behavior unacceptable; from slightly to extremely (*n* = 1018). The mean acceptability score for the pooled sample is 2.35 (*SD* = 1.73), indicating that most women found the behavior moderately unacceptable on average. Those who previously engaged in the foodie call behavior perceived this behavior significantly more acceptable (*M* = 4.65, *SD* = 1.62) than those who didn’t (*M* = 2.50, *SD* = 1.62), *t* = 26.8, *p* = .000, *d* = 1.32. Table 5 and Figure 2 show the distribution of the acceptability data in detail.

**Table 5.** Frequencies of Foodie Call Acceptability.

	Acceptability Ratings									
	Cluster	1	2	3	4	5	6	7	Total	Avg Ratings
Original study	USA	253	141	72	71	71	47	19	674	2.68
Current study	PL	141	116	102	106	40	38	4	547	2.85
	UK	166	71	29	18	16	2	6	308	1.95
	USA	202	106	85	177	179	144	90	983	3.83
Total of current		509	293	216	301	235	184	100	1838	2.35

*Note*. 1 = extremely unacceptable, 2 = moderately unacceptable, 3 = slightly unacceptable, 4 = neutral, …, 7 = extremely acceptable.

### Structural Equation Models

**Figure 3.**
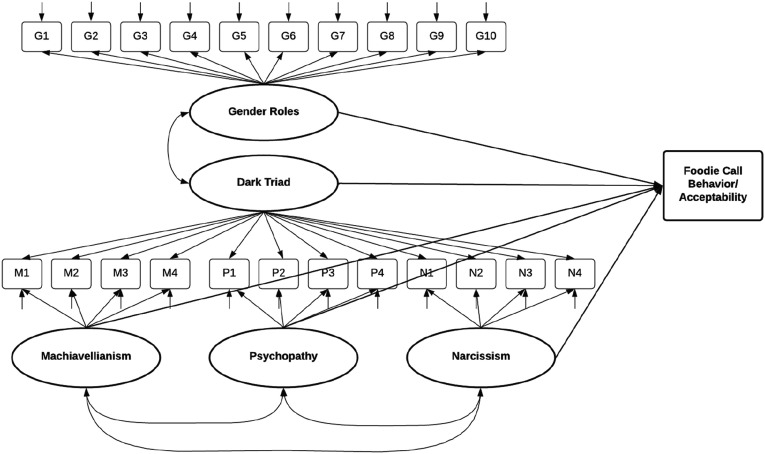
Study model. Note: In Model 1, the foodie call behavior is the dependent variable. In Model 2, it is its acceptability.

To follow the replication procedure, we tested the same models of the original study represented in Figure 3. The first model was related to the behavior itself, and the second model estimated its acceptability, of which both are predicted by gender role beliefs, each individual dimension of the Dark Triad traits (Machiavellianism, psychopathy, and narcissism), and their general factor, which is the constellation of the Dark Triad traits. As illustrated in the Figure 3, the Dark Triad traits were inserted into the analyses as a bifactor model, assuming that not only individual traits, but also the general factor of all three dark traits could predict the behavior and its acceptability. The confirmatory factor analysis of the bifactor model resulted in an acceptable fit; root mean square error approximation (RMSEA) = .077, 95% CI [.075, .080], Comparative Fit Index (CFI) = .996, Tucker-Lewis Index (TLI) = .995, and Standardized Root Mean Square Residual (SRMR) = .049. The standardized estimates for the paths predicting the behavior and its acceptability are presented in Table 6 and Table 7 respectively.

**Table 6.** Structural Equation Model Examining Whether the Dark Triad and Gender Roles Predict Previous Foodie Call Behavior (Pooled Sample, *N* = 1838).

Measure	Estimate	SE	*β*	z	*p*
Dark triad general factor	.194	.13	.174	1.46	.145
Machiavellianism	−.061	.12	−.005	−.49	.621
Psychopathy	−.030	.15	−.011	−.20	.841
Narcissism	.189	.07	.079	2.62	.009
Gender role beliefs	.883	.22	.616	4.04	<.001

**Table 7.** Structural Equation Model Examining Whether the Dark Triad and Gender Roles Predict the Acceptability (Pooled Sample, *N* = 1838).

Measure	Estimate	SE	*β*	z	*p*
Dark triad general factor	.104	.11	.093	.96	.339
Machiavellianism	−.042	.10	−.003	−.41	.679
Psychopathy	−.319	.12	−.120	−2.61	.009
Narcissism	.223	.06	.093	3.57	<.001
Gender role beliefs	1.051	.18	.732	5.84	<.001

To test structural equation models in estimating the foodie call behavior and its acceptability, we used the exact syntax code provided by the original paper’s authors. The estimations used in both models were based on diagonally weighted least squares (DWLS), which produced robust statistics along with robust standard errors. The optimization method in all models was Nonlinear Minimization subject to Box Constraints (NLMINB), which is the default option for the lavaan package. No other estimation model other than DWLS was converged.

Both models confirmed that traditional gender role beliefs were the strongest predictor of the behavior and its acceptability (see Tables 6 and 7). In addition, narcissism was found to be another significant predictor of the behavior, even though the effect was found relatively smaller (*β* = .079, *p* = .009), but the general factor of the Dark Triad was not significant. The same conclusion was drawn for acceptability, such that the Dark Triad traits failed to predict acceptability. However, two subdimensions of the Dark Triad were related to acceptability. As predicted, the narcissism construct is positively associated with acceptability (*β* = .093, *p* = .000). Surprisingly, psychopathy was negatively predictive of perceived foodie call acceptability based on the pooled sample data. Detailed analyses are available via OSF.

In addition to the analyses applied to the pooled sample, we ran the same models at the country level. The fit statistics of models tested for each country are illustrated in Table 8. The models based on the US and Polish samples indicated a good fit. The poorest fit statistics were observed in the UK sample; nevertheless, CFI and TFI figures were above acceptable levels. Rigdon (1996) and Chen et al. (2008) favored the use of CFI over RMSEA in exploratory studies. Therefore, we proceeded with the models under the assumption that they secured satisfactory levels of fit statistics. Table 9 shows the estimates for the models tested in our Polish, UK, and US samples respectively.

**Table 8.** Goodness of Fit Statistics of Tested Models at the Country Level.

Cluster	Poland (*n* = 547)		UK (*n* = 308)		USA (*n* = 983)	
	DV = Behavior	DV = Acceptability	DV = Behavior	DV = Acceptability	DV = Behavior	DV = Acceptability
RMSEA	.066	.066	.097	.098	.064	.065
RMSEA 95% CI	[.061, .071]	[.061, .071]	[.090, .104]	[.091, .105]	[.061, .068]	[.061, .069]
CFI	.981	.981	.975	.974	.998	.998
TFI	.977	.977	.970	.969	.997	.997
SRMR	.069	.068	.096	.095	.044	.044

**Table 9.** Structural Equation Models.

	Polish Sample (*n* = 547)									
Measure	DV = Behavior					DV = Acceptability				
	Estimate	SE	*β*	z	*p*	Estimate	SE	*β*	Z	*p*
Dark triad general factor	.321	.09	.279	3.64	<.001	.307	.07	.266	4.73	<.001
Machiavellianism	−.012	.72	—	−.018	.986	−.344	.60	—	−.57	.568
Psychopathy	.036	.10	.028	.36	.722	.100	.07	.076	1.42	.157
Narcissism	.181	.09	.142	1.97	.049	.200	.06	.157	3.52	<.001
Gender role beliefs	.439	.18	.281	2.45	.014	.079	.15	.051	.54	.590
	UK Sample (*n* = 308)									
Measure	DV = Behavior					DV = Acceptability				
	Estimate	SE	*β*	z	*p*	Estimate	SE	*β*	Z	*p*
Dark triad general factor	.276	.13	.251	2.10	.036	.432	.06	.392	6.89	<.001
Machiavellianism	.083	.63	—	.13	.894	.519	.37	—	1.41	.158
Psychopathy	−.003	.22	−.002	−.01	.989	.154	.17	.084	.90	.370
Narcissism	.291	.12	.210	2.52	.012	.127	.08	.092	1.62	.106
Gender role beliefs	.524	.31	.266	1.69	.091	−.027	.24	−.014	−.11	.912
	UK Sample (*n* = 983)									
Measure	DV = Behavior					DV = Acceptability				
	Estimate	SE	*β*	z	*p*	Estimate	SE	*β*	Z	*p*
Dark triad general factor	.174	.24	.156	.73	.466	−.023	.16	−.021	−.15	.885
Machiavellianism	−.540	.33	—	−1.63	.103	−.215	.23	—	−.94	.345
Psychopathy	.307	.28	.086	1.12	.265	−.238	.17	−.066	−1.44	.150
Narcissism	.301	.14	.109	2.09	.036	.264	.11	.095	2.44	.015
Gender role beliefs	.690	.36	.473	1.93	.054	1.206	.23	.825	5.23	<.001

The Polish sample replicated the exact effects as reported originally. The previous foodie calls were predicted by the general factor of the Dark Triad (*β* = .279, *p* = .036) and gender role beliefs (*β* = .281, *p* = .014). However, the acceptability of the behavior was best predicted by the common factor of dark personality (*β* = .266, *p* = .000). Additionally, narcissism appears to be the only Dark Triad trait predicting acceptability in the Polish sample (*β* = .157, *p* = .000). Some of these results are also relevant for the UK sample, replicating the initial effect partially. The acceptability of foodie call behavior was predicted by the common Dark Triad factor (*β* = .392, *p* = .000), and no other variable predicted the acceptability in the UK. Previous foodie calls was not predicted by gender role beliefs. Instead, narcissism (*β* = .210, *p* = .012) and the general factor (*β* = .251, *p* = .036) best predicted the behavior. These results, using the UK sample, also partially confirmed the replicability of the original findings. Finally, the US sample revealed the most surprising results in our study because neither the general factor of the Dark Triad nor gender role belief significantly predicted foodie calls. Even though gender role beliefs were found to have a relatively higher effect size, the effect was insignificant at the .05 level (*β* = .473, *p* = .054). Still, the behavior could be predicted by narcissism (*β* = .109, *p* = .036). The strongest predictor of the foodie call acceptability among the US women was gender role beliefs (*β* = .825, *p* = .000), followed by narcissism (*β* = .095, *p* = .015). One common characteristic to all sample’s analyses was that Machiavellianism failed to predict foodie calls and their acceptability, even though the original finding showed a significant effect.

## Discussion

In efforts to replicate and generalize the original findings, our study revealed mixed results. Collisson et al. (2020) found that the general factor of the Dark Triad (Machiavellianism, Psychopathy, and Narcissism) predicted women’s foodie call behavior and its acceptability. Our results indicated that gender role beliefs were the strongest predictor of foodie calls and their perceived acceptability, with the general Dark Triad traits not being significant. At the country level, the Polish sample replicated the effects of Dark Triad and gender role beliefs on foodie calls, and the UK sample partially replicated these findings. However, gender roles did not seem to affect the behavior of UK women. The US sample also partially replicated the original findings, with gender role beliefs strongly associated with acceptability and the general factor of Dark Triads failing to predict foodie calls or their acceptability. Surprising findings emerged in our study as foodie calls were more common and acceptable in the US than previously reported. In our sample, 52.8% of US women engaged in foodie calls, while the reported frequencies in the original study were 23% and 33%. These differences suggest potential changes in foodie call trends in the US dating context. Additionally, our data failed to support the general factor of dark personality traits predicting foodie call behavior and its acceptability, indicating a lack of replication in the US.

Our study found cultural differences in foodie calls and their acceptability. We offer several explanations as to why we found differences. As hypothesized during pre-registration, we claimed that if traditional gender roles are explicit in a society, women would presumably not pay for a meal when dating. Consequently, irrespective of Dark Triad traits, women may enjoy a free meal when asked out on a date. That is, traditional gender roles may partly explain why women engage in foodie calls. Nevertheless, the Dark Triad findings were replicated in the UK and Poland. Regarding gender inequality, the UK and Poland exhibit different cultural characteristics, so gender roles alone could not be the sole explanation for less frequent foodie calls. Other factors, such as socio-economic conditions, individual-level personality traits, and cultural norms, may also contribute to these differences. Even though not directly assessed in our study, future studies can control for economic and social variables to evaluate the impact of such conditions. Additionally, other personality traits at the individual level, and differences in cultural norms, such as the level of masculinity in society, may be influential.

Another possible explanation for the partial replication may be changing trends in US dating. In the past 2 years since the original findings were published, they have garnered a great deal of popular media attention, particularly within the US (e.g., via national television on Good Morning America and the Daily Show with Trevor Noah). As more American women learned about foodie calls, they may perceive foodie calls as more common and acceptable. Widespread media attention could lead to *availability cascade*, “a self-reinforcing process of collective belief formation by which an expressed perception triggers a chain reaction that gives the perception increasing plausibility through its rising availability in public discourse” (Kuran & Sunstein, 1999, *p*.683). Thus, such behavior becomes not only more prevalent but can be considered more acceptable through aggrandized public discourse. Also, familiarity with the concept may remove the naivety of subjects who participated in this study. Chandler et al. (2015) empirically established that including non-naïve subjects in studies can reduce effect sizes in psychological studies. As a result, due to the changing public discourse, and familiarity with the foodie call behavior, the Dark Triad may fall short in predicting the behavior and its acceptability in the US.

Moreover, the original study collected data in 2018, prior to the COVID-19 pandemic and economic inflation concerns. To the extent to which US dating norms may have changed as a result of COVID-19 and economic concerns, such as women dating men to whom they are not attracted to combat loneliness and financial worries, perhaps foodie calls are becoming more common. It is important to note that any failure to replicate, or in our case, partially replicated findings, may reveal a false positive in the original study, a false negative in the replication study, or reveal meaningful moderators between the conditions of the original and replication studies.

Although our pre-registered replication had many strengths, such as its cross-cultural replication of a timely dating phenomenon, it also had limitations which warrant discussion. For instance, our study compared foodie calls among women in Poland, the UK, and the US. Additional comparison groups, especially large, representative samples, may further extend our findings and reveal meaningful moderating variables. Moreover, we were also bound by the cross-sectional, online survey design, which relied on self-report methods. Accurately assessing Dark Triad traits, for example, is a challenge in online research. Future studies which assess foodie calls and individual differences using peer-report and other nuanced methods may be worthwhile. Finally, it should be noted that ‘foodie call’ is only associated with women in the existing literature, and the behavior and its acceptability are very low in different cultures, as presented in our study. Thus, findings of this study should be also interpreted with caution. To understand the complex mechanisms involved in dating, future studies can include all genders and unpack the global impact of Dark Triad personality traits on relational exchanges.

## References

[bibr1-00332941231164079] Arends-Tóth J. van de Vijver F. J. R. (2007). Cultural and gender differences in gender-role beliefs, sharing household task and child-care responsibilities, and well-being among immigrants and majority members in The Netherlands. Sex Roles, 57(11–12), 813–824. 10.1007/s11199-007-9316-z.

[bibr2-00332941231164079] Boehnke M. (2011). Gender role attitudes around the globe: Egalitarian vs. traditional views. Asian Journal of Social Science, 39(1), 57–74. 10.1163/156853111X554438

[bibr3-00332941231164079] Brandt M. J. IJzerman H. Dijksterhuis A. Farach F. J. Geller J. Giner-Sorolla R. Grange J. A. Perugini M. Spies J. R. Van't Veer A. (2014). The replication recipe: What makes for a convincing replication? Journal of Experimental Social Psychology, 50, 217–224. 10.1016/j.jesp.2013.10.005

[bibr16-00332941231164079] Brown M. J. Gladstone N. (2012). Development of a short version of the gender role beliefs scale. International Journal of Psychology and Behavioral Sciences, 2(5), 154–158. 10.5923/j.ijpbs.20120205.05.

[bibr4-00332941231164079] Campbell W. K. Foster J. D. (2007). The narcissistic self: Background, an extended agency model, and ongoing controversies. In Sedikides C. Spencer S. J. (Eds), Frontiers of social psychology. The self (pp. 115–138). Psychology Press.

[bibr5-00332941231164079] Chandler J. Paolacci G. Peer E. Mueller P. Ratliff K. A. (2015). Using nonnaive participants can reduce effect sizes. Psychological Science, 26(7), 1131–1139. 10.1177/0956797615585115 26063440

[bibr6-00332941231164079] Chen F. Curran P. J. Bollen K. A. Kirby J. Paxton P. (2008). An empirical evaluation of the use of fixed cutoff points in RMSEA test statistic in structural equation models. Sociological Methods and Research, 36(4), 462–494. 10.1177/0049124108314720 19756246 PMC2743032

[bibr7-00332941231164079] Collisson B. Howell J. L. Harig T. (2020). Foodie calls: When women date men for a free meal (rather than a relationship). Social Psychological and Personality Science, 11(3), 425–432. 10.1177/1948550619856308

[bibr8-00332941231164079] Czarna A. Z. Jonason P. K. Dufner M. Kossowska M. (2016). The dirty dozen scale: Validation of a polish version and extension of the nomological net. Frontiers in Psychology, 7, 445. 10.3389/fpsyg.2016.00445 27065915 PMC4811972

[bibr9-00332941231164079] Delucia J. L. (1987). Gender role identity and dating behavior: What is the relationship? Sex Roles, 17(3–4), 153–161. 10.1007/BF00287622

[bibr10-00332941231164079] Eagly A. H. (1987). Sex differences in social behavior: A social-role interpretation. Erlbaum.

[bibr11-00332941231164079] Eaton A. A. Rose S. (2011). Has dating become more egalitarian? A 35 year review using sex roles. Sex Roles, 64(11–12), 843–862. 10.1007/s11199-011-9957-9

[bibr12-00332941231164079] Food and Agricultural Organization . (2022). World food situation: Global price index. https://www.fao.org/worldfoodsituation/foodpricesindex/en/

[bibr13-00332941231164079] Gillespie B. J. Peterson G. Lever J. (2019). Gendered perceptions of fairness in housework and shared expenses: Implications for relationship satisfaction and sex frequency. PloS ONE, 14(3), Article e0214204. 10.1371/journal.pone.0214204 30893363 PMC6426245

[bibr14-00332941231164079] Going Dutch . (2022). In Wikipedia. https://en.wikipedia.org/wiki/Going_Dutch

[bibr15-00332941231164079] Jaramillo-Sierra A. L. Allen K. R. (2013). Who pays after the first date? Young men’s discourses of the male-provider role. Psychology of Men and Masculinity, 14(4), 389–399. 10.1037/a0030603

[bibr17-00332941231164079] Jonason P. K. Webster G. D. (2010). The dirty dozen: A concise measure of the dark triad. Psychological Assessment, 22(2), 420–432. 10.1037/a0019265 20528068

[bibr18-00332941231164079] Kerr P. S. Holden R. R. (1996). Development of the gender role beliefs scale (GRBS). Journal of Social Behavior and Personality, 11(5), 3–16. 10.1037/t14862-000

[bibr19-00332941231164079] Klein R. A. Cook C. L. Ebersole C. R. Vitiello C. Nosek B. A. Hilgard J. Ahn P. H. Brady A. J. Chartier C. R. Christopherson C. D. Clay S. Collisson B. Crawford J. T. Cromar R. Gardiner G. Gosnell C. L. Grahe J. Hall C. Howard I. ... Ratliff K. A. (2022). Many Labs 4: Failure to replicate mortality salience effect with and without original author involvement. Collabra: Psychology, 8(1), 35271. 10.1525/collabra.35271

[bibr20-00332941231164079] Klein R. A. Ratliff K. A. Vianello M. Adams R. B. Jr. Bahník Š. Bernstein M. J. Bocian K. Brandt M. J. Brooks B. Brumbaugh C. C. Cemalcilar Z. Chandler J. Cheong W. Davis W. E. Devos T. Eisner M. Frankowska N. Furrow D. Galliani E. M. ... Nosek B. A. (2014). Investigating variation in replicability: A “many labs” replication project. Social Psychology, 45(3), 142–152. 10.1027/1864-9335/a000178

[bibr21-00332941231164079] Kuran T. Sunstein C. R. (1999). Availability cascades and risk regulation. Stanford Law Review, 51(4), 683–768. 10.2307/1229439

[bibr22-00332941231164079] Lever J. Frederick D. A. Hertz R. (2015). Who pays for dates? Following versus challenging gender norms. SAGE Open. 10.1177/2158244015613107

[bibr23-00332941231164079] Lucas A. (2022). Rapidly rising food prices may give restaurants an edge—here’s why. CNBC. https://www.cnbc.com/2022/01/12/rapidly-rising-food-prices-may-give-restaurants-an-edgeheres-why.html

[bibr24-00332941231164079] Miller J. D. Lynam D. R. Hyatt C. S. Campbell W. K. (2017). Controversies in narcissism. Annual Review of Clinical Psychology, 13, 291–315. 10.1146/annurev-clinpsy-032816-045244 28301765

[bibr25-00332941231164079] Paulhus D. L. Williams K. M. (2002). The dark triad of personality: Narcissism. Machiavellianism, and psychopathy. Journal of Research in Personality, 36(6), 556–563. 10.1016/S0092-6566(02)00505-6

[bibr26-00332941231164079] Rigdon E. E. (1996). CFI versus RMSEA: A comparison of two fit indexes for structural equation modeling. Structural Equation Modeling: A Multidisciplinary Journal, 3(4), 369–379. 10.1080/10705519609540052

[bibr27-00332941231164079] Rudman L. A. Glick P. (2021). The social psychology of gender: How power and intimacy shape gender relations (2nd ed.). The Guilford Press.

[bibr28-00332941231164079] Simonsohn U. (2015). Small telescopes: Detectability and the evaluation of replication results. Psychological Science, 26(5), 559–569. 10.1177/0956797614567341 25800521

[bibr29-00332941231164079] Stavrova O. Fetchenhauer D. Schlösser T. (2012). Cohabitation, gender, and happiness: A cross-cultural study in thirty countries. Journal of Cross-Cultural Psychology, 43(7), 1063–1081. 10.1177/0022022111419030

[bibr30-00332941231164079] Swanson (2022, February 3) Food prices approach record highs, threatening the world’s poorest. The New York Times. https://www.nytimes.com/2022/02/03/business/economy/food-prices-inflation-world.html

[bibr31-00332941231164079] The World Bank . (2022). GDP per capita (current US$) - Poland, United Kingdom, United States. https://data.worldbank.org/indicator/NY.GDP.PCAP.CD?locations=PL-GB-US

[bibr32-00332941231164079] United Nations Development Programme . (2020). Gender inequality index (GII). https://hdr.undp.org/data-center/thematic-composite-indices/gender-inequality-index#/indicies/GII

[bibr33-00332941231164079] Vogel D. L. Wester S. R. Heesacker M. Madon S. (2003). Confirming gender stereotypes: A social role perspective. Sex Roles, 48(11/12), 519–528. 10.1023/A:1023575212526

[bibr34-00332941231164079] Wu H. Luo S. Klettner A. White T. Albritton K. (2021). Gender roles in the millennium: Who pays and is expected to pay for romantic dates? Psychological Reports. 00332941211057144. Advance online publication. 10.1177/00332941211057144 34874209

